# Effectiveness of Plasma-Treated Hydrogen Peroxide Mist Disinfection in Various Hospital Environments

**DOI:** 10.3390/ijerph18189841

**Published:** 2021-09-18

**Authors:** Jongbong Choi, Minhyuk Lee, Yangsoon Lee, Yeongtak Song, Yongil Cho, Tae Ho Lim

**Affiliations:** 1 Department of Biomedical Engineering, Hanyang University, Seoul 04763, Korea; cjbonghyu@gmail.com (J.C.); alsgur1529@gmail.com (M.L.); 2 Department of Laboratory Medicine, College of Medicine, Hanyang University, Seoul 04763, Korea; yangsoon@hanyang.ac.kr; 3 Department of Emergency Medicine, College of Medicine, Hanyang University, Seoul 04763, Korea; yeongtaksong@hanyang.ac.kr (Y.S.); joeguy@hanmail.net (Y.C.)

**Keywords:** plasma activation, hydrogen peroxide, surface disinfection, hospital rooms, surface type

## Abstract

Hospital environments are associated with a high risk of infection. As plasma-treated hydrogen peroxide mist disinfection has a higher disinfection efficacy, we tested the efficacy of plasma-treated hydrogen peroxide mist disinfection on several surfaces in various hospital environments. Disinfection was performed in 23 rooms across different hospital environments, including hospital wards, outpatient departments (OPDs), and emergency rooms. A total of 459 surfaces were swabbed before/after disinfection. Surfaces were also divided into plastic, metal, wood, leather, ceramic, silicone, and glass for further analyses. Only gram-positive bacteria were statistically analyzed because the number of gram-negative bacteria and mold was insufficient. Most colony-forming units (CFUs) of gram-positive bacteria were observed in OPDs and on leather materials before disinfection. The proportion of surfaces that showed a percentage decrease in CFU values of more than 90% after disinfection were as follows: OPDs (85%), hospital wards (99%), and emergency rooms (100%); plastic (97%), metal (83%), wood (84%), leather (81%), and others (87%). Plasma-treated hydrogen peroxide mist disinfection resulted in a significant decrease in the CFU values of gram-positive bacteria in various environments. Plasma-treated hydrogen peroxide mist disinfection is an effective and efficient method of disinfecting various hospital environments.

## 1. Background

Recently, the importance of efficient and effective disinfection in preventing infection has been emphasized worldwide due to the impact of Coronavirus disease (COVID-19) [1]. Generally, gram-positive bacteria (*Staphylococcus*, *Clostridium*, *Mycobacterium*, etc.), gram-negative bacteria (*Acetobacter*, *Escherichia*, *Pseudomonas*, *Salmonella*, *Serratia*, etc.), and mold (*Aspergillus*, *Trichophyton*, etc.) carry the risk of infection. Moreover, hospital environments are associated with a high risk of infection with antibiotic-resistant bacteria such as methicillin-resistant *Staphylococcus aureus* (MRSA), vancomycin-resistant *Enterococcus* (VRE), and carbapenem-resistant *Enterobacteriaceae* (CRE). Therefore, effective solutions to prevent transmission between infected and non-infected patients are needed in medical facilities and hospitals. Several studies have reported that devices such as patient bed rails and blood pressure cuffs in hospitals are contaminated by bacteria, including both pathogens and non-pathogens [2,3,4,5,6,7]. Although disinfection of surfaces and equipment that may come into contact with patients is important for preventing cross-infection, such measures are difficult to carry out appropriately with limited human resources. Disinfecting a wide area that requires repeated management by human resources is not easy, and complete disinfection efficacy would not be guaranteed.

Disinfectants such as hydrogen peroxide and chlorine-based sodium hypochlorite are commonly used in hospital settings, in which various disinfection methods such as dry fogging and mist-spraying are employed. Hydrogen peroxide (H_2_O_2_) is less harmful to patients than other agents, as only water (H_2_O) and oxygen (O) remain after decomposition. In addition, hydrogen peroxide exhibits a high disinfection efficacy for infectious bacteria; thus, disinfection equipment (Sterinis^®^ (STERIS Corporation, Mentor, OH, USA), Nocospray^®^ (OXY’PHARM, Champigny-sur-Marne, France), Bioquell^®^,Q-10 (Bioquell, Andover, UK) and Deprox^®^ (Hygiene Solutions, Kings Lynn, UK)) that uses hydrogen peroxide as a disinfectant is widely utilized, and several studies have reported that hydrogen peroxide can inactivate pathogens [8,9,10,11]. In addition, plasma can achieve effective disinfection by generating ions, electrons, active species, electric fields, and ultraviolet radiation, with some studies reporting the inactivation of bacteria [12,13]. Highly reactive species such as O, OH, and NO_2_ are generated by cold plasma and play the most crucial role in inactivating microorganisms, while ultraviolet plays a secondary role [14]. Both hydrogen peroxide and the generated reactive species (O, OH, and NO_2_) inactivate microorganisms. Also 2nd activation reactants such as H_2_O_2_ combined with H_2_O in hydrogen peroxide and O in reactive species could increase the disinfection efficacy, resulting in higher disinfection efficacy than the use of hydrogen peroxide alone. Previous research has indicated that plasma-treated hydrogen peroxide is associated with a 6-log change in disinfection efficacy [15].

Despite the advantage of plasma-treated hydrogen peroxide mist disinfection methods, the effectiveness of disinfection in various hospital environments has rarely been analyzed. In this study, we aimed to compare cultured bacteria and mold before and after disinfection to verify the efficacy of plasma-treated hydrogen peroxide mist disinfection for inactivating bacteria and mold on surfaces in various hospital environments. Additionally, we would like to suggest efficient infection control based on the disinfection characteristics results.

## 2. Materials and Methods

### 2.1. Experiment

The study was conducted at a university hospital (855 beds, 2500 outpatients per day) in Seoul, Korea. The guidelines for disinfection in hospitals are to wipe with chlorine-based disinfectants and use a higher concentration in situations where there is a risk of infection. The study was conducted after disinfection according to the general disinfection management guidelines in the hospital. Disinfection was performed in 23 hospital rooms, including seven hospital wards, 12 outpatient departments (OPDs), and four emergency rooms. The selected hospital wards were patient ward rooms for one and four patients: normal isolation rooms, VRE isolation rooms, isolation rooms in the intensive care unit (ICU), dialysis isolation rooms, and peritoneal dialysis rooms. OPDs included the computed tomography (CT) room; X-ray room; tuberculosis (TB) examination room; infectious disease (ID) examination room; paediatric (PED) examination room; ear, nose, throat (ENT) examination room; ophthalmology (OT) examination room; dental (DENT) examination room; chest medicine (CM) endoscopy room; gastroenterology (GE) endoscopy room; general surgery (GS) endoscopy room; obstetrics and gynecology (OBGY) delivery room. Emergency rooms included the resuscitation room, paediatric room, critical care room, and triage room.

Frequently touched devices and surfaces by the patient and medical staff in the hospital environments were selected. A range from a minimum of 15 to a maximum of 24 surfaces per room (*n* = 459 total surfaces) were swabbed and cultured before and after disinfection in 23 rooms across different hospital environments. Objects used as cultured surfaces included beds, telephones, desks, chairs, cabinets, knobs, electric devices, and medical devices. In addition, to compare the disinfection efficacy based on the material comprising the objects, the 459 surfaces were divided into 275 plastic materials, 85 metal materials, 50 wooden materials, 28 leather materials, 17 ceramic materials, three silicone materials, and one glass material.

**Figure 1:**
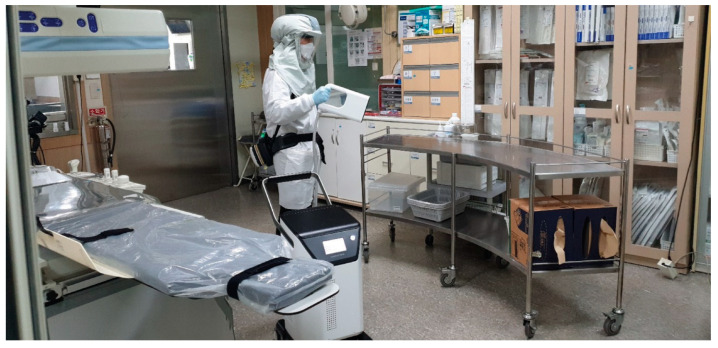
Plasma-treated hydrogen peroxide mist disinfection in a hospital environment using a PlaClin^®^ disinfector and disinfectant containing 5.9% w/w hydrogen peroxide.

For disinfection, the doors and windows of the selected rooms were all closed and non-sealed, and disinfectant was sprayed for from approximately 1 to 3 s to ensure that the mist touched each surface sufficiently. In this study, we used PlaClin^®^ plasma-treated hydrogen peroxide mist surface disinfector (CODESTERI Inc, Seoul, Korea) and PlaClinSol^®^ disinfectant (CODESTERI Inc., Seoul, Korea) containing 5.9% w/w (weight/weight) hydrogen peroxide and undisclosed additional substances (Figure 1). For the safety monitoring, the concentration of hydrogen peroxide in the air inside and outside the 23 rooms was measured in real-time using Polytron 7000^®^ hydrogen peroxide detectors (Draeger, Lübeck, Germany). All bacteria on the surfaces were cultured before and after disinfection using swabs. To identify both opportunistic and pathogenic bacteria, swab samples were inoculated on 5% sheep blood agar and MacConkey agar and incubated at 37 °C for 24 h. Gram staining was performed on the cultured bacterial colonies. The representative species was identified by matrix-assisted laser desorption/ionization-time of flight mass spectrometry (MALDI-TOF MS) with a MALDI Biotyper using MALDI-Biotyper software (version 2.3, Bruker Daltonics, Bremen, Germany). Bacterial identification was performed by an experienced laboratory medical specialist.

### 2.2. Statistical Analysis

Statistical analysis was performed using the SAS version 9.4 (SAS Institute Inc., Cary, NC, USA). The Wilcoxon rank-sum test was used to identify the statistical significance of cultured bacteria among surfaces and materials before disinfection, and the Wilcoxon signed-rank test was used to determine whether disinfection significantly decreased the number of bacteria relative to that observed prior to disinfection. The colony-forming unit (CFU) of bacteria represented the variable used for statistical analysis. Study data were collected from August 2019 to January 2021.

## 3. Results

### 3.1. Cultured Surfaces Positive for Microorganisms

As shown in Table 1, we classified microorganisms cultured from 459 surfaces into gram-positive or gram-negative bacteria and molds. Gram-positive bacteria were classified into bacillus and cocci, and positive bacillus and cocci cultures were observed for 185 and 326 surfaces before disinfection, respectively, and 41 and 16 surfaces after disinfection, respectively. The total number of cultured-positive surfaces for gram-positive bacteria was 353, because surfaces positive for both bacillus and cocci were counted as a single instance. In addition, gram-negative bacteria were classified into rods, bacilli, and cocci. The culture-positive numbers for gram-negative bacteria were three, one, and one surfaces before disinfection, respectively, and only one surface was culture-positive for rod bacteria after disinfection. Thirty-four surfaces were positive for mold prior to disinfection, although no surfaces were positive for mold after disinfection. Given the low number of gram-negative bacteria and mold specimens identified, data were analyzed for gram-positive bacteria only. Some bacterial species were identified using the MALDI-TOF MS. The representative gram-positive bacilli were identified as belonging to the genus *Bacillus,* including *B. cereus*, *B. infantis*, *B. megaterium*, *B simples*, and *B. circulans*, *Paenibacillus glucanolyticus*, and the genus *Streptomyces*. The representative gram-positive cocci were identified as coagulase-negative *Staphylococci* (*S. hominis* and *S. capitis*), *Staphylococcus aureus*, *Micrococcus luteus,* and *Kocuria rhizophilisa*. The gram-negative bacilli were identified as *Acinetobacter* sp. and *Pantoea* sp.

### 3.2. Disinfection Efficacy (23 Hospital Rooms)

As shown in Table 2, the median CFU values (Q_1_–Q_3_) of gram-positive bacteria in hospital wards, OPDs, and emergency rooms before disinfection were 1 (0–7), 7 (2–26), and 5 (1–28), respectively. After disinfection, these values were all 0 (0–0). After disinfection, the median percentage decreases (range) in hospital wards, OPDs, and emergency rooms were 100% (0–100), 100% (−500–100), and 100% (0–100), respectively. The proportion of sampled surfaces that showed decrease rates of more than 90% were 99%, 85%, and 100% in hospital wards, OPDs, and emergency rooms, respectively (samples where CFU = 0 before disinfection were excluded).

We further examined the significance of differences in CFU before disinfection among the 23 rooms. The *p*-values for the differences in CFU before disinfection between the corresponding room and other rooms were *p* < 0.0001 for hospital wards, *p* < 0.0001 for OPDs, and *p* = 0.2015 for emergency rooms, respectively, and most CFUs were observed in OPDs, followed by emergency rooms and hospital wards. Differences in CFU values before and after disinfection for each of the 23 rooms were significant.

### 3.3. Disinfection Efficacy for Seven Types of Surfaces

In the present study, the 459 surfaces were classified into seven materials: plastic, metal, wood, leather, ceramic, silicon, and glass. Three materials (ceramic, silicon, and glass) were included in the experimental group because the number of materials was not sufficient for statistical analysis. As shown in Table 3, the median CFU values (IQR) of gram-positive bacteria in the plastic, metal, wood, leather, and other subgroups before disinfection were 4 (1–22), 4 (1–16), 3.5 (0–11), 9.5 (4.5–23.5), and 2 (0–4), respectively. Moreover, these values were all 0 (0–0) after disinfection, except for leather, which had values of 0 (0–0.5). After disinfection, the median percentage decreases (range) for plastic, metal, wood, leather, and other materials were 100% (−400 to 100), 100% (−500 to 100), 99% (−20 to 100), 100% (−29 to 100), and 100% (0–100), respectively. Decreases of more than 90% in the plastic, metal, wood, leather, and other subgroups were noted in 97%, 83%, 84%, 81%, and 87% of cases, respectively, when cases where CFU = 0 before disinfection were excluded.

We also investigated the statistical significance of differences in CFU values before disinfection among the seven material groups. The *p*-values for the difference in CFU values before disinfection among the materials were as follows: plastic (*p* = 0.7776), metal (*p* = 0.7233), wood (*p* = 0.1499), leather (*p* = 0.0199), and other (*p* = 0.0399). The highest CFU value was observed for the leather subgroup, followed by the plastic, metal, wood, and other subgroups. The differences in CFU before and after disinfection for each of the seven materials were statistically significant (*p* < 0.0001).

## 4. Discussion

Previous studies have highlighted the importance of hand-touched surfaces in the transmission of pathogens to healthy people and patients with various medical conditions [16,17]. In addition, research has indicated that surfaces closer to the patient are associated with a greater risk of infection than those farther away [18,19]. In the present study, we compared the levels of cultured bacteria and mold before and after disinfection to verify the efficacy of plasma-treated hydrogen peroxide mist disinfection and mold on surfaces in various hospital environments. We observed more gram-positive bacterial CFUs in OPDs than in hospital wards and emergency rooms prior to disinfection in 23 rooms, suggesting that the CFUs are increased because patients touch more surfaces in OPDs than in other environments. In particular, the CFU value for the ophthalmic examination room was the highest among OPDs, possibly because there are many ophthalmic examination devices with which patients come into direct contact. On the other hand, the isolation rooms exhibited the lowest CFU values, likely due to the routine application of infection control for a high risk of infection such as CRE and VRE.

Our findings indicated that 85% of OPDs exhibited a percentage decrease of more than 90% after disinfection, which was relatively lower than that observed for hospital wards (99%) and emergency rooms (100%). Ledwoch et al. reported that biofilms containing bacterial pathogens are virtually universal on hospital surfaces and that they are more formed by numerous gram-positive bacteria [20]. In the case of OPDs, we believe numerous biofilms were formed because the CFU values for gram-positive bacteria were relatively higher than in the hospital ward and emergency room before/after disinfection in our study. Simões et al. reported that cleaning is the most importance first step because disinfectants do not completely penetrate the biofilm matrix, could not destroy all the living biofilm cells [21]. These findings suggest that, prior to disinfection, mechanical cleaning may be needed to remove and reduce dirt, debris, and other organic matter such as blood, secretions, and excretions [22].

Our findings also indicated that the highest CFU values for gram-positive bacteria occurred on leather surfaces. This is because leather can provide a high-moisture environment and a suitable temperature for bacterial growth and rapid colonization, leading to the formation of a biofilm [23,24,25,26]. Gough et al. reported that disinfection is more readily carried out on plastic than on wooden surfaces [27] and that plasma-treated hydrogen peroxide mist disinfection also appears to be more effective for disinfecting plastic (97%) than all other materials (81% to 87%).

This study has several limitations. First, the presence or absence of cleaning and cleaning methods prior to disinfection was not the same for each room. However, we believe that these conditions appropriately reflect the situation in actual hospital environments. Second, since the median percentage decrease included cases in which CFU = 0 before disinfection, it was difficult to compare these decreases. Therefore, cases in which CFU = 0 before disinfection were excluded when analyzing percentage decreases of more than 90% to ensure a reliable comparison of disinfection efficacy among hospital rooms or surfaces. Third, since this was a single-center study, it is difficult to generalize the results. In the future, repeated multicenter studies are required to verify our findings.

Otter et al. reported that surfaces contaminated by gram-positive bacteria contribute to the transmission of pathogens [28]. After the plasma-treated hydrogen peroxide mist disinfection of surfaces in various hospital environments, we observed significant decreases in the CFU values of gram-positive bacteria. However, we were unable to statistically analyze data for gram-negative bacteria and mold due to the small number of CFUs. Although the CFU values of gram-negative bacteria and mold were not sufficient, percentage decreases of more than 90% were observed in 100% of cases for all culture-positive hospital rooms and surfaces.

Our results further suggest that plasma-treated hydrogen peroxide mist disinfection can be performed without sealing the rooms because it did not spread outside, even without sealing, when monitored with a hydrogen peroxide detector. Moreover, it took an average of one hour to reach 1 ppm, the Occupational Safety and Health Administration (OSHA) safety regulation for hydrogen peroxide after disinfection for all 23 rooms without ventilations. Appropriate disinfection can be performed with limited human resources and time because mobile medical devices and equipment that require disinfection can be collected and disinfected at the same time. In addition, hydrogen peroxide is environmentally friendly because only water and oxygen remain after decomposition.

Abreu et al. divided disinfectants into four levels (low, intermediate, high, and sterilants), hydrogen peroxide corresponds to high-level disinfectant, and higher concentrations correspond to sterilant levels [29]. Additionally, there are some studies about disinfection using airborne hydrogen peroxide in vapor or dry-mist formulations, and hydrogen peroxide has been reported to cause the inactivation of bacterial spores as an additional benefit to conventional mechanical cleaning regimens [30]. Furthermore, despite several reports of verification of disinfection efficacy according to the disinfection method, studies on field tests under practical conditions were rarely reported [31]. In this study, we used plasma-treated hydrogen peroxide mist for sterilant-level disinfection, not hydrogen peroxide only. We also tried to verify the disinfection efficacy with field tests under practical conditions.

## 5. Conclusions

In conclusion, although several studies have investigated the efficacy of various disinfectants and disinfection methods, few studies have conducted such an analysis based on different surface types in different hospital environments. In this study, we verified the disinfection efficacy of plasma-treated hydrogen peroxide mist disinfection in various hospital rooms and for various types of surfaces. Ultimately, we believe that utilizing the disinfection characteristics of various rooms and surface types will help to promote efficient infection control.

## Figures and Tables

**Table 1:** Surfaces positive for bacteria and mold before and after disinfection with plasma-treated hydrogen peroxide mist disinfectant.

		Number of Culture-Positive Surfaces [Total: 459]	
		Before Disinfection	After Disinfection
**Bacteria**	**Gram (+)**	353 [459] *	57 [459] *
	Bacillus (+)	185	41
	Cocci (+)	326	16
	**Gram (−)**	**5 [459]**	**1 [459]**
	Rod (−)	3	1
	Bacillus (−)	1	0
	Cocci (−)	1	0
**Mold**	**Mold**	**34 [459]**	**0 [459]**
**Total**		**392 [459]**	**58 [459]**

* In the gram-positive bacteria count, bacillus (+) and cocci (+) were cultured together and counted as one surface when dual positivity was observed.

**Table 2:** CFU values of gram-positive bacteria from surfaces in 23 rooms (three types) before and after disinfection with plasma-treated hydrogen peroxide mist disinfectant.

Room	No. of Culture Positive Surfaces [Total Surfaces]	CFU, Median (IQR)		CFU after Disinfection		Difference in CFU, *p*-Value	
		Before Disinfection	After Disinfection	% Decrease, Median (Range)	>90% Decrease, % *	Before Disinfection †	Before-After Disinfection ‡
**Hospital ward**	**88 [151]**	**1 (0–7)**	**0 (0–0)**	**100 (0–100)**	**99**	**<0.0001**	**<0.0001**
1 patient	17 [20]	10.5 (3–31.5)	0 (0–0)	100 (0–100)	100	0.0744	<0.0001
4 patients	18 [22]	9.5 (2–34)	0 (0–0)	100 (0–100)	100	0.1113	<0.0001
Isolation	08 [20]	0 (0–3)	0 (0–0)	0 (0–100)	100	0.0002	0.0078
Isolation (VRE)	11 [23]	0 (0–2)	0 (0–0)	0 (0–100)	100	<0.0001	0.0010
Isolation (ICU)	12 [18]	2 (0–4)	0 (0–0)	100 (0–100)	100	0.0483	0.0005
Isolation (Dialysis)	10 [24]	0 (0–3)	0 (0–0)	0 (0–100)	91	0.0002	0.0010
Peritoneal Dialysis	12 [24]	0.5 (0–2.5)	0 (0–0)	50 (0–100)	100	0.0005	0.0005
**OPD**	**191 [219]**	**7 (2–26)**	**0 (0–0)**	**100 (−500–100)**	**85**	**<0.0001**	**<0.0001**
CT	13 [15]	4 (1–83)	0 (0–1)	100 (0–100)	77	0.4791	0.0005
X-Ray	16 [16]	7 (2.5–65)	0 (0–1)	100 (67–100)	88	0.0820	<0.0001
Examining (TB)	14 [14]	13.5 (7–25)	0 (0–0)	100 (75–100)	93	0.0133	0.0001
Examining (ID)	14 [16]	10.5 (2.5–19)	0 (0–1)	97 (−500–100)	79	0.3177	0.0037
Examining (PED)	22 [24]	7 (1.5–38)	0 (0–1)	100 (−29–100)	73	0.1275	<0.0001
Examining (ENT)	16 [18]	16.5 (5–34)	0 (0–0)	100 (0–100)	88	0.0423	<0.0001
Examining (OT)	15 [15]	100 (32–103)	0 (0–0)	100 (91–100)	100	<0.0001	<0.0001
Examining (DENT)	13 [16]	5 (1–16.5)	0 (0–2)	95 (−400–100)	69	0.9062	0.0039
Endoscopy (CM)	15 [21]	2 (0–10)	0 (0–0)	100 (0–100)	100	0.2672	<0.0001
Endoscopy (GE)	20 [24]	3 (1–8.5)	0 (0–0)	100 (0–100)	85	0.3569	<0.0001
Treatment (GS)	18 [20]	7 (3–16)	0 (0–1)	100 (0–100)	72	0.1651	<0.0001
Delivery (OBGY)	15 [20]	3 (1–9)	0 (0–0)	100 (0–100)	100	0.4717	<0.0001
**Emergency room**	**74 [89]**	**5 (1–28)**	**0 (0–0)**	**100 (0–100)**	**100**	**0.2015**	**<0.0001**
Resuscitation	16 [23]	1 (0–5)	0 (0–0)	100 (0–100)	100	0.0370	<0.0001
Pediatric	22 [24]	24 (5.5–38.5)	0 (0–0)	100 (0–100)	100	0.0015	<0.0001
Critical care	18 [24]	3.5 (0.5–55.5)	0 (0–0)	100 (0–100)	100	0.5536	<0.0001
Triage	18 [18]	3.5 (3–13)	0 (0–0)	100 (100–100)	100	0.5263	<0.0001
**Total**	**353 [459]**	**4 (1–20)**	**0 (0–0)**	**100 (−500–100)**	**92**		**<0.0001**

* When CFU = 0 before disinfection, the value was excluded from analysis of >90% decrease. † The *p*-value is the difference in CFU before disinfection between the corresponding room and other rooms (Wilcoxon rank-sum test). ‡ The *p*-value is the difference in CFU before and after disinfection in each room (Wilcoxon signed-rank test). Abbreviations: CFU: colony-forming unit; VRE: vancomycin-resistant *Enterococcus*; ICU: intensive care unit; TB: tuberculosis; ID: infectious disease; PED: pediatrics; ENT: ear, nose, and throat; OT: ophthalmology; DENT: dental; CM: chest medicine; GE: gastroenterology; GS: general surgery; OBGY: obstetrics and gynecology; IQR: interquartile range.

**Table 3:** CFU values of gram-positive bacteria from seven types of materials before and after disinfection with plasma-treated hydrogen peroxide mist disinfector.

Material	No. of Culture Positive Surfaces [Total Surfaces]	CFU, Median (IQR)		CFU after Disinfection		Difference in CFU, *p*-Value	
		Before Disinfection	After Disinfection	% Decrease, Median (Range)	>90% Decrease, % *	Before Disinfection †	Before-After Disinfection ‡
Plastic	209 [275]	4 (1–22)	0 (0–0)	100 (−400–100)	97	0.7776	<0.0001
Metal	71 [85]	4 (1–16)	0 (0–0)	100 (−500–100)	83	0.7233	<0.0001
Wood	31 [50]	3.5 (0–11)	0 (0–0)	99 (−20–100)	84	0.1499	<0.0001
Leather	27 [28]	9.5 (4.5–23.5)	0 (0–0.5)	100 (−29–100)	81	0.0119	<0.0001
Etc. §	15 [21]	2 (0–4)	0 (0–0)	100 (0–100)	87	0.0399	<0.0001
**Total**	**353 [459]**	**4 (1–20)**	**0 (0–0)**	**100 (−500–100)**	**92**		**<0.0001**

* When CFU = 0 before disinfection, values were excluded from the analysis of >90% decrease. † The *p*-value is the difference in CFU before disinfection between the corresponding room and other rooms (Wilcoxon rank-sum test). ‡ The *p*-value is the difference in CFU before and after disinfection in each room (Wilcoxon signed-rank test). § Ceramics, silicon, and glass are included. The number of culture-positive surfaces (total surfaces) was 11 [17], three [3], and one [1], respectively. Abbreviations: CFU: colony-forming unit; IQR: interquartile range.
